#  Intestinal Obstruction due to Ileal Duplication Cyst and Malrotation in a Preterm Neonate 

**Published:** 2015-10-01

**Authors:** Laxman Basany, Roja Aepala, Madhu Mohan Reddy Bellary, Maruthi Chitneni

**Affiliations:** 1 Department of Neonatology, Dolphin Children's Hospital, Hyderabad, Telangana, India; 2 Department of Pediatric Surgery, Dolphin Children's Hospital, Hyderabad, Telangana, India

**Dear Sir**

The baby was born to a 24-year-old primigravida mother by cesarean section at 36 week gestation and weighed 2420 grams. Antenatal scan at 32 week gestation showed dilated bowel loops and polyhydramnios (Amniotic fluid index: 32 cm) suggestive of intestinal obstruction. He developed respiratory distress soon after birth and was referred to our unit. On examination, the baby had respiratory distress with RR of 72/min. and sub costal retractions. Abdomen was distended at birth and baby had 120 ml of bilious aspirate in the first 14 hours of life. X-ray of abdomen showed dilated proximal bowel loops with absence of gas in distal bowel loops suggestive of intestinal obstruction. A provisional diagnosis of intestinal atresia was made. Laparotomy showed duplication cyst measuring 6 cm in diameter along the antimesenteric border involving proximal ileum and malrotation of small bowel (Fig. 1) Duplication cyst along with adjoining portion of ileum measuring 13.5 cm was resected and end to end anastomosis was done. Ladd’s procedure was done for malrotation. Histopathology showed the cyst attached to serosal aspect on the anti-mesenteric side without communication with ileum, but had a common muscular wall lined with simple columnar epithelium consistent with ileal duplication cyst. Baby was fed from the 7th day and was discharged uneventfully on the 14th postoperative day. 

**Figure:**
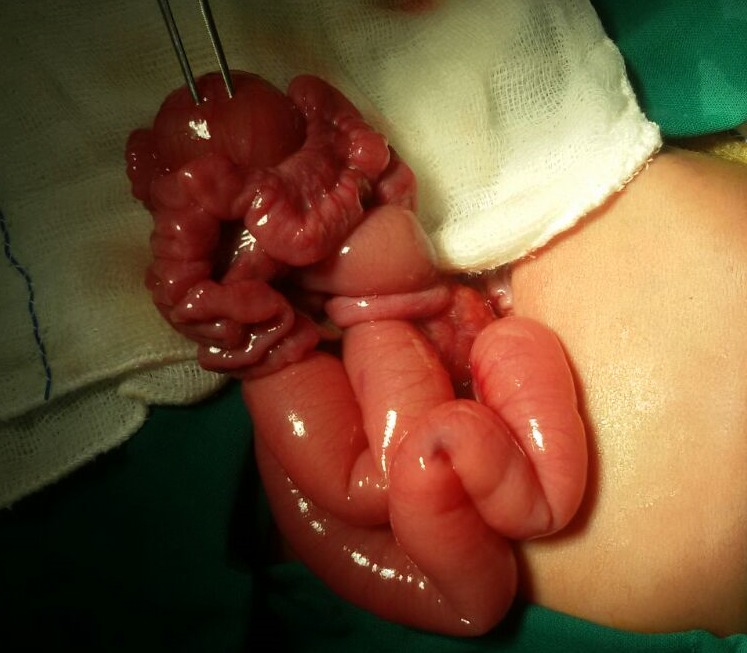
Figure 1: Cystic ileal duplication cyst.

Enteric duplication cysts (EDCs) are very rare with an estimated incidence of 1 in 4500 births. EDCs may be suspected by sonographic demonstration of an intra-abdominal cystic mass in the second or third trimester of gestation. The differential diagnosis of antenatal intra-abdominal cysts includes ovarian cysts, enteric duplication cysts, renal cysts, choledochal cysts, hepatic cysts, mesenteric or omental cysts and dilated bowel loops of intestinal atresia.[1] Sonographic findings highly suggestive of an intestinal origin of the cyst are peristaltic muscular contractions of the cyst wall, double layered wall and intimate contact with the mesenteric border.[1-3] Prenatal diagnosis of EDC is often difficult and sonography identifies 20 to 30% of lesions. In our case, antenatal scan showed dilated bowel loops and polyhydramnios suggestive of intestinal obstruction but duplication cyst was not noticed.

EDCs can give rise to symptoms at any age but usually present during infancy with varied clinical presentation, depending on the location, size and the presence of heterotopic gastric mucosa. [1-3] Heterotopic gastric mucosa can cause bleeding or perforation by peptic ulceration. Enteric duplications can serve as the focal point for volvulus or intussusceptions.[4] EDC can be asymptomatic and the diagnosis is often made incidentally during a surgical procedure. 

Resection of the duplication cyst along with the adjacent bowel is suggested owing to the possibility of malignant changes and the risk of gastrointestinal obstruction, ulceration and hemorrhage due to ectopic gastric mucosa.[4] Large duplications, however, are difficult to resect due to the risk of short-bowel syndrome. Mucosal stripping offers a good surgical option in these cases, eliminating the possibility of subsequent peptic ulceration or carcinogenesis. According to the proposed vascular classification, resection of the duplication cyst without the adjacent bowel seems possible in some cases.[4] In our case, resection of EDC along with adjacent portion of ileum was performed as it was not possible to separate the ileum from the duplication cyst.

## Footnotes

**Source of Support:** Nil

**Conflict of Interest:** None
